# Perioperative Management of Paediatric Hypertension

**DOI:** 10.3390/children12091174

**Published:** 2025-09-03

**Authors:** Nicole Barbosa, Cara Redelinghuys, Palesa Mogane

**Affiliations:** 1 Department of Anaesthesia, School of Clinical Medicine, Charlotte Maxeke Johannesburg Academic Hospital University of the Witwatersrand, Johannesburg 2193, South Africa; 2 Department of Anaesthesia, School of Clinical Medicine, Chris Hani Baragwaneth Academic Hospital, University of the Witwatersrand, Johannesburg 1864, South Africa; carared003@gmail.com (C.R.); palesa.mogane@wits.ac.za (P.M.)

**Keywords:** perioperative, paediatric, hypertension

## Abstract

Paediatric hypertension presents significant perioperative challenges due to its variable aetiology and potential for end-organ damage. The prevalence varies with age and is associated with both primary and secondary causes, which differ markedly from adult hypertension. This review outlines the classification, diagnosis, and causes of paediatric hypertension to provide context for its management in the perioperative setting. Emphasis is placed on the identification and preoperative optimisation of hypertension, intraoperative blood pressure control, and the management of hypertensive crises. Specific perioperative strategies, including anaesthetic planning, pharmacological interventions, and postoperative monitoring, are discussed. Specific conditions such as phaeochromocytoma and aortic coarctation require tailored pharmacological strategies and close interdisciplinary collaboration. Postoperative care in an intensive care setting is essential for monitoring complications and achieving long-term blood pressure control. Effective perioperative management of paediatric hypertension requires early identification, thorough preoperative assessment, and prompt intraoperative and postoperative intervention. Multidisciplinary care and an understanding of paediatric specific pathophysiology are key to reducing morbidity and improving outcomes.

## 1. Introduction and Prevalence

Hypertension is increasingly recognised in paediatric patients, with a prevalence of 2–5% [1]. In neonates, the prevalence is lower, where 1–1.8% are affected [2,3]; however, data from the AWAKEN (Assessment of Worldwide Acute Kidney Injury Epidemiology in Neonates) cohort study revealed that 3.7% of newborns had undiagnosed hypertension [3]. Estimates of the actual prevalence of hypertension in the paediatric population are complicated by the lack of normal data for paediatric blood pressure [1,2,4,5,6]. Additionally, the use of antihypertensive medications in paediatric patients younger than six years has not been approved by regulatory agencies [2,3,4,7]. While this review includes relevant background on the epidemiology and aetiology of paediatric hypertension, the focus remains on perioperative considerations, particularly during hypertensive crises and surgical optimisation.

### 1.1. Definition of Chronic Hypertension in Paediatric Patients

Hypertension in neonates is defined as a blood pressure (BP) measurement above the 95th percentile for post-menstrual age (Table 1) [4,8]. This definition is not derived from study results but rather extrapolated from the definition used in older children [4,8]. In children aged < 13 years, the definition of hypertension is based on percentiles derived from sex, age, and height [1]. In children over 13 years of age, the definitions and staging of hypertension are guided by the 2017 American Heart Association (AHA) and American College of Cardiology guidelines for adults [1].

The challenges in defining hypertension in children are posed by jurisdictional differences between American and European Guidelines. American guidelines classify BP as normal, elevated, stage 1, and stage 2 hypertension, based on percentiles as see in Table 2 [1], while European guidelines define BP as normal, high-normal, and hypertensive, with distinctions between the stages of severity based on the presence of target organ damage or other risk factors [8].

Dione et al. [4] recommend using frequency distribution curves to guide management decisions, stating that regular BP measurements above the 95th percentile should be monitored closely, and those above the 99th percentile should be investigated and potentially treated, depending on clinical signs and symptoms. These recommendations were supported by the 2017 American Academy of Paediatrics (AAP) Paediatric Hypertension [1].

### 1.2. Measuring Blood Pressure in Paediatric Patients

The recommended protocol requires measuring BP at least three times at different visits, with an appropriate technique, which may be auscultatory using a sphygmomanometer or oscillometric, using a paediatric approved, calibrated device [1]. Before the measurement, the child should be seated in a quiet room. For consistency, the right arm should be used unless there is abnormal aortic arch anatomy. It is essential to use a properly sized cuff with a bladder length covering 80–100% of the arm’s circumference and a width of at least 40% [1].

For children in whom determining the appropriate cuff size is challenging, midarm circumference should be measured. This is performed by identifying the midpoint between the acromion of the scapula and olecranon of the elbow while keeping the shoulder in a neutral position, and the elbow flexed at 90°. This ensures the accurate selection of the correct cuff size [1]. If the initial reading exceeds the appropriate 90th percentile for the patient, two additional auscultatory measures should be taken, and the average of the three systolic and diastolic readings should be used to determine the child’s BP category [1].

## 2. Aetiology of Chronic Hypertension in Paediatric Patients

In contrast to adults, where hypertension is frequently primary or idiopathic, the cause of hypertension in children varies significantly with age and is often secondary to other pathologies. Primary hypertension in children usually occurs after the age of 6 years and is associated with a family history of hypertension, obesity, and sedentary lifestyle [1,9].

Secondary causes of hypertension in paediatric patients are summarised in Table 3 [4,10,11,12]. The most common secondary causes of hypertension include renal, renovascular, endocrine, neurological and medication related factors. While the clinical presentation varies with age, these conditions often require similar diagnostic approaches and perioperative considerations.

## 3. Secondary Causes of Chronic Hypertension in Paediatric Patients

Renal and Renovascular

The common cause of renal and renovascular hypertension in neonates include renal artery thrombus development (often secondary to the placement of an umbilical artery catheter), polycystic kidney disease and congenital obstructive uropathies [12,13]. In older children and adolescents, renal parenchymal diseases, such as glomerulonephritis, and renovascular disease, such as Takayasu arteritis and fibromuscular dysplasia are important considerations [14].

Endocrine

Endocrine causes include congenital adrenal hyperplasia due to 11β hydroxylase or 17α hydroxylase deficiency, Cushing’s syndrome, hyperaldosteronism, and hyperthyroidism [1,4,12].

Neurological

Seizures, intracranial hypertension and pain cause episodic hypertension and require appropriate management [4,12].

Pulmonary

Neonatal hypertension is commonly caused by chronic lung disease, with bronchopulmonary dysplasia (BPD) being a significant contributor [12].

Cardiovascular

Coarctation of the aorta initially causes upper limb hypertension, which may persist after surgical correction [12,15].

Medications

Corticosteroids, bronchodilators, and vasopressors can elevate BP and perioperative planning should account for these exposures [4,8]. In adolescents, non-adherence to antihypertensives or substance abuse, such as amphetamines or cocaine, should also be considered.

Neoplasia

Intra-abdominal and extra-abdominal tumours such as neuroblastoma, Wilms lymphoma, phaeochromocytomas, and paragangliomas can be associated with hypertension, which can be due to direct compression of renal vessels and ureters, and to the production of vasoactive catecholamines. Although rare, pheochromocytomas and paragangliomas can be fatal [7,16].

Genetic factors

Several genetic syndromes are known to predispose children to hypertension, often through their association with endocrine or renal pathologies [2,10,17]. These genetic factors may play a crucial role in the development of hypertension and ischemic heart disease in adulthood [18].

## 4. Perioperative Management of Severe Hypertension

A hypertensive crisis refers to a significant, abrupt rise in BP from baseline, which may cause end-organ damage and be life threatening.

There are two categories: hypertensive emergency and hypertensive urgency.

Hypertensive emergency is defined by a severe elevation in blood pressure associated with acute end-organ damage, requiring immediate treatment [11].

Hypertensive urgency presents with significantly elevated blood pressure without end-organ damage, and treatment can proceed more gradually and often presents with non-specific signs, such as lethargy and irritability, in neonates and infants. In older children, the symptoms may include nausea, headaches, dizziness, palpitations, syncope, and fatigue [11,14].

Signs and symptoms of hypertensive emergencies:

Neurological:

Seizures can cause cerebral haemorrhage or infarction. Fundoscopy should always be performed as it may reveal papilloedema, retinal haemorrhage, and exudates (which may be the only clinical signs of impending hypertensive encephalopathy) [11].

Cardiovascular:

Signs and symptoms of tachypnea, hypoxia, pulmonary edoema and the presence of a cardiac gallop or ‘S3’ on auscultation are indicative of left ventricular failure [11].

Renal:

Renal manifestations of hypertensive crisis can include acute kidney injury, as well as haematuria and proteinuria [11].

### 4.1. Preoperative Optimisation for Anaesthesia

Preoperative optimisation of hypertension is important to prevent further end-organ damage, such as cardiovascular instability, stroke, and renal dysfunction [11]. A comprehensive preoperative assessment should include screening for pre-existing hypertension and evaluation for potential secondary causes. Recognising these factors help guide perioperative blood pressure management and may reduce the risk of complications. The American Academy of Paediatrics (AAP) guidelines emphasise that secondary causes of hypertension should be identified and corrected prior to surgery when possible, and that antihypertensive medication should be continued throughout the perioperative period [19]. The American Heart Association (AHA) suggests that a delay of 1–6 weeks may be appropriate before surgery to evaluate and manage hypertension in certain time-sensitive procedures, a recommendation based on adult guidelines and therefore this recommendation must be cautiously extrapolated when applied to children [20]. Risk benefit discussions should involve the paediatrician, anaesthetist and surgeon, and patients should be reviewed before elective surgery. Accurate measurement of BP is essential to avoid factors such as pain, anxiety, fear, and stress that cause transient hypertension [21].

### 4.2. Intraoperative Management of Hypertensive Emergencies

Intraoperative hypertensive crises may arise suddenly in paediatric patients with known or undiagnosed hypertension, requiring immediate recognition and stabilisation. Changes in BP can be sudden and unpredictable; therefore, non-invasive BP monitoring every 1–2 min or continuous invasive pressure monitoring is recommended for at-risk patients [1].

Intravenous access (IV), either by means of a reliable peripheral line or central venous catheter, is required for the administration of antihypertensive agents. Monitoring vital organ function by means of electrocardiography (ECG), pulse oximetry, capnography, and temperature are important. A urinary catheter should be used to measure urine output, and cardiac output measurement via transthoracic or transesophageal echocardiography may be useful in patients with cardiovascular instability [22].

Maintaining intraoperative cardiovascular stability is challenging and often requires pharmacological support in conjunction with reliable anaesthetic management [22]. The anaesthetic management of choice should avoid catecholamine release, which may be triggered by medications (e.g., ephedrine, pethidine, ketamine, and metoclopramide), anaesthetic procedures (e.g., tracheal intubations), or surgical stimulation (surgical incision, abdominal exploration, tumour manipulation, and increased intra-abdominal pressures caused by laparoscopic procedures) [11,23]. This can be achieved through pre-emptive analgesia, smooth induction, and maintenance of adequate anaesthetic depth.

**Figure 1:**
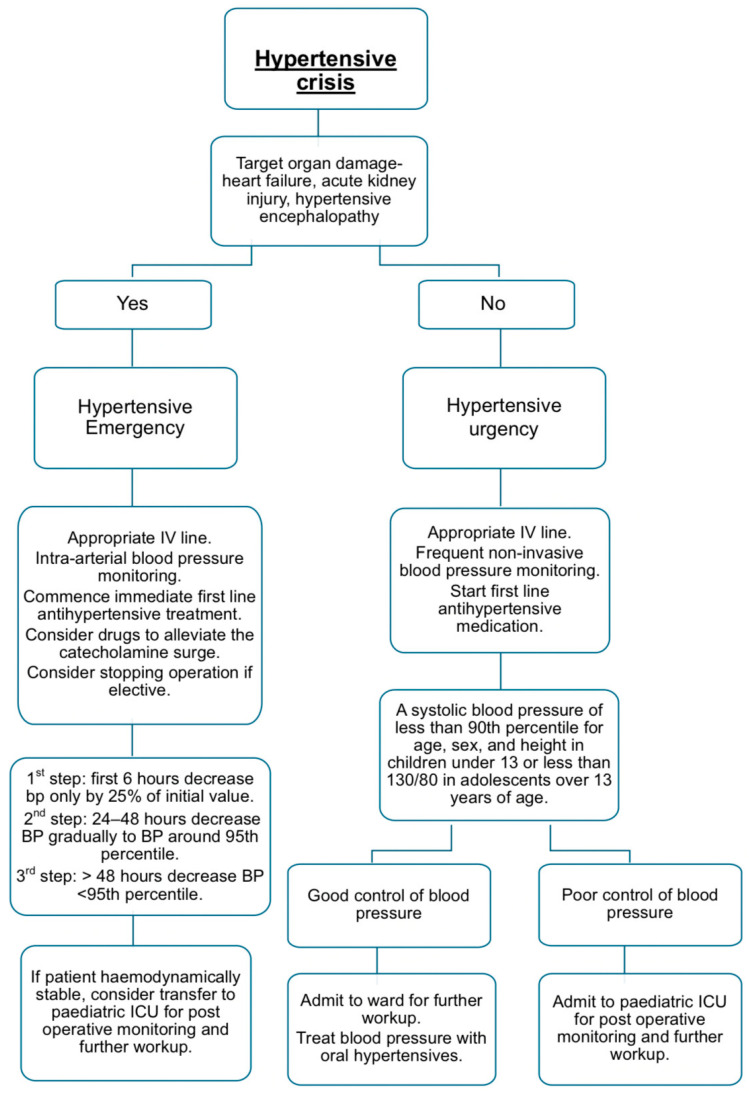
Flow diagram for the management of hypertensive crisis [11,14].

In cases of intraoperative or postoperative hypertension, a thorough evaluation and adjustment of anaesthesia and analgesia is critical. If hypertension persists despite adequate anaesthetic depth, or if a secondary cause is suspected, the decision to initiate antihypertensive treatment should balance the risk of rapid BP reduction against the risks associated with sustained hypertension. Antihypertensive agents should be introduced cautiously and titrated to avoid precipitating hypotension. A structured approach to the management of hypertensive crises is attached (Figure 1) [11,14].

### 4.3. Pharmacological Management of Intraoperative Hypertensive Crises

Careful use of anaesthetic agents, including opioids and rapidly acting antihypertensive agents, is essential to prevent serious morbidity and mortality during perioperative hypertensive crises [24]. During an intraoperative hypertensive crisis, close communication between the surgical and anaesthesia teams is crucial for anticipating and managing problems.

Due to the absence of specific paediatric guidelines, treatment is based on adult protocols [1,11,25], and BP should be reduced by 25% over a 6–8 h period by using continuous infusions of short-acting antihypertensive agents [14]. For children under 13 years, the ideal systolic BP is less than the 90th percentile for age, sex, and height, whereas for adolescents over 13 years, it should be below 130/80 mmHg [25]. Continuous infusions can be adjusted until the target BP is achieved, according to the patient’s response. Sudden drops in BP should be avoided, as they can result in cardiac and cerebral ischemia or haemorrhage [1,26].

Paediatric patients with chronic hypertension are at greater risk of relative hypotension and poor cerebral perfusion if their BP rapidly normalises perioperatively [21]. This is due to the loss of autoregulatory tone following prolonged hypertension. In contrast, paediatric patients with normal BP who experience an acute intraoperative hypertensive episode are less likely to have an increased cerebrovascular autoregulatory threshold and can tolerate rapid BP normalisation [21]. The treatment goal should be personalised for each patient based on their response to treatment and, if known, the underlying cause of hypertension [1].

There are limited Federal Drug Administration (FDA)-approved drugs for the treatment of severe hypertension in paediatric patients, with only half of the currently available antihypertensive drugs sold in the United States of America labelled for paediatric use, often resulting in common off-label use by medical practitioners [25].

Remifentanil is a mu-opioid receptor agonist with a rapid onset and offset that is effective in reducing hemodynamic responses. While not studied specifically in hypertensive children, the use of remifentanil has been shown to control the hypertensive response to intubation in paediatric patients up to 12 years of age, the recommended IV dose is 2–3 µg/kg bolus over 30–60 s, or as an infusion at 0.1–0.5 µg/kg/min [23,27].

Magnesium sulphate has been demonstrated to block the release of catecholamines from the adrenal medulla and peripheral adrenergic nerve terminals. It acts as an antagonist of L-type calcium channels, producing antiarrhythmic actions and decreasing alpha-adrenergic receptor sensitivity to catecholamines. Additionally, magnesium directly dilates the arteriolar vessels. A dose of 30 mg/kg IV over 10 min could be administered to attenuate the hypertensive response [23,28].

Dexmedetomidine is a sedative and analgesic that selectively binds to alpha-2 receptors in the central nervous system, inhibiting sympathetic activity and reducing BP and heart rate. The recommended loading dose is 1 µg/kg IV over 10–30 min, followed by an infusion of 0.2–0.7 µg/kg/h [23,28]. If dexmedetomidine is unavailable, clonidine (alpha-2 receptor agonist) can be used as an alternative, at a dose of 2–5 µg/kg per dose, up to 10 µg/kg, every 6–8 h via a nasogastric tube [1].

The 2017 AAP Paediatric Hypertension Guidelines recommend the use of esmolol, hydralazine, nicardipine, labetalol, and nitroprusside as first-line agents for patients with life-threatening symptoms due to hypertensive emergencies [1,26]. A summary of the most used antihypertensive agents in intraoperative hypertensive emergencies is provided in Table 4.

#### 4.3.1. First-Line Agents

##### Vasodilators

Nitroprusside is one of the most prescribed medications for paediatric hypertensive crises owing to its rapid onset and offset. It is effective in treating both hypertension and congestive heart failure because it induces venous and arterial vasodilation, reducing the preload and afterload. However, prolonged use (>24–48 h) may lead to thiocyanate toxicity, which may result in seizures, metabolic acidosis, or methemoglobinemia [11].

Hydralazine is a direct arteriolar vasodilator that decreases systemic vascular resistance and consequently lowers BP. However, common side effects include reflex tachycardia, activation of the renin–angiotensin–aldosterone system, and salt retention, especially when the medication does not counteract the negative inotropic effects on the heart [11,14].

##### Adrenergic Blockers and Agonists

Esmolol is a short-acting cardioselective beta-1 adrenergic blocker. It is commonly used in paediatric hypertensive crises, although adverse effects, such as wheezing, hypotension, and bradycardia, can occur. At higher doses, esmolol may lose its beta-1 selectivity and activate beta-2 receptors in bronchioles, potentially causing bronchoconstriction [11,14].

##### Calcium Channel Blocker

Nicardipine is a second-generation dihydropyridine calcium channel blocker (CCB) that causes peripheral vasodilation and relaxation of vascular smooth muscle cells. It is commonly used in paediatric patients with severe hypertension. The main side effects included palpitations, flushing, and tachycardia [11,14].

#### 4.3.2. Second-Line Agents

##### Vasodilators

Diazoxide is a direct vasodilator that facilitates potassium movement across smooth muscle membranes and effectively lowers BP in paediatric patients at lower doses. However, they are not available in many countries [11].

Nitroglycerin (also known as glyceryl trinitrate) is a potent vasodilator and nitric oxide donor that reduces preload and cardiac output, although it is rarely used as a first-line treatment [11,14].

##### Adrenergic Blockers and Agonists

Labetalol, which combines alpha-1 and beta-adrenergic blockade, lowers BP by reducing peripheral vascular resistance, with minimal impact on cardiac output. However, it can cause bronchoconstriction and negative inotropic effects, rendering it unsuitable for patients with asthma or unstable congestive heart failure. During the first six hours of treatment, labetalol has been shown to significantly lower the BP [11,14].

Phentolamine is a competitive, non-selective alpha-receptor antagonist that leads to vasodilation but may cause reflex tachycardia. Its short half-life of 19 min makes it useful for setting target infusion rates during hypertensive surgery. However, tachyphylaxis can also occur repeatedly [23].

##### Angiotensin-Converting Enzyme (ACE) Inhibitors

Enalaprilat is an IV ACE inhibitor recommended for paediatric patients with high renin hypertension, renovascular hypertension, or reno-parenchymal hypertension. It can cause hyperkalemia and acute kidney injury. Before initiating enalaprilat therapy, renal Doppler ultrasound is advised to exclude bilateral renal artery stenosis or stenosis of a single kidney, as it is contraindicated in these cases. This recommendation is based on adult literature and expert consensus, given the risk of precipitating acute kidney injury ACE inhibitors should be used cautiously in paediatric patients with volume depletion because their elevated renin levels can lead to hypotension [11,14].

##### Diuretics

Furosemide (a loop diuretic) can be effective for paediatric patients with volume-dependent hypertension, such as those with glomerulonephritis, congestive heart failure, or oliguric acute kidney injury. It lowers the BP by initiating natriuresis and diuresis. However, it is crucial to monitor serum potassium levels and hydration status regularly, as repeated use can result in hypokalemia or volume depletion [11].

### 4.4. Management of Hypertensive Crisis in Specific Conditions

#### 4.4.1. Phaeochromocytoma

In patients with phaeochromocytoma, alpha-adrenergic antagonists like phentolamine are the recommended first-line medications [23]. Once full alpha blockade is achieved, beta-blockers such as esmolol can be added if tachycardia is present. Beta-blockers should not be used alone without alpha-blockers, as this may cause unopposed alpha-adrenergic activation, leading to a further increase in BP. Calcium channel blockers may be considered as second- or third-line therapies for paediatric patients with pheochromocytoma [14,23].

#### 4.4.2. Aortic Coarctation

Paediatric patients with aortic coarctation often require beta-adrenergic medications such as esmolol to manage BP. Esmolol is also beneficial after coarctectomy because patients can experience paradoxical hypertension following repair. Care must be taken when lowering BP in these patients, as a significant drop may result in the hypoperfusion of critical organs. Ideally, these patients should be managed by a cardiac anaesthetist or an anaesthetist experienced in this field [11,14].

## 5. Postoperative Care

Successful perioperative management of paediatric hypertension hinges on identifying high-risk patients early, ensuring appropriate anaesthetic preparation, and using targeted pharmacological interventions guided by multidisciplinary input.

The decision to extubate after surgery is influenced by hemodynamic stability and management of other critical factors. Postoperative monitoring in an intensive care unit is required to detect potential complications. Invasive arterial pressure monitoring should be performed for at least 24 h post-surgery. Following surgery, hypertension may result from pain, urinary retention, fluid overload, and unintentional renal artery occlusion, which cause hyperreninemia, incomplete tumour excision, or metastatic illness [22]. Paediatric patients with isolated hypertension that resolves rapidly and shows no evidence of target organ damage do not typically require further workup or management.

The duration of BP control after initiating treatment depends on various patient-specific factors, such as the presumed duration of the hypertensive episode, necessary interventions, and underlying cause. It is advisable to refer patients for further diagnostic assessments and consultation with paediatric specialists, including paediatric nephrologists or intensivists, for appropriate ongoing management [1,11,25].

## 6. Conclusions

Optimal perioperative management of paediatric hypertension requires a comprehensive understanding of its underlying causes, age-specific definitions, and the physiological impact of elevated blood pressure. Hypertensive crises in children require prompt recognition and treatment to prevent severe organ damage [11].

While general principles of paediatric hypertension are important, perioperative care demands tailored approaches to preoperative optimisation, intraoperative monitoring, and pharmacological control of hypertensive crises. Anaesthetists must collaborate closely with paediatricians, nephrologists, and surgeons to minimise the risk of end-organ damage and haemodynamic instability [23].

Long-term management of paediatric hypertension is critical, especially for those with chronic hypertension. If the underlying cause of hypertension is successfully treated, then acute BP control is usually sufficient. However, chronic hypertension can lead to serious long-term cardiovascular and renal consequences that require ongoing monitoring and intervention. Successful perioperative management can improve outcomes and prognosis; however, careful attention to the potential for persistent hypertension is necessary to prevent further complications in these vulnerable patients.

## Figures and Tables

**Table 1:** Systolic, mean, and diastolic BP for infants after 2 weeks of life by post-menstrual age [4,8].

Postmenstrual Age (Weeks)	50th Percentile SBP/DBP (MAP)	95th Percentile SBP/DBP (MAP)	99th Percentile SBP/DBP (MAP)
26	55/38 (30)	72/57 (50)	77/63 (56)
28	60/45 (38)	75/58 (50)	80/63 (54)
30	65/48 (40)	80/63 (55)	85/68 (60)
32	65/48 (40)	83/64 (55)	88/69 (60)
34	70/50 (40)	85/65 (55)	90/70 (60)
36	72/57 (50)	87/72 (65)	92/77 (70)
38	77/59 (50)	92/74 (65)	97/79 (70)
40	80/60 (50)	95/75 (65)	100/80 (70)
42	85/62 (50)	98/76 (65)	102/81 (70)
44	88/63 (50)	105/80 (68)	110/85 (73)

SBP = Systolic Blood Pressure; DBP = Diastolic Blood Pressure; MAP = Mean Arterial Pressure.

**Table 2:** Definitions of BP Categories and Stages: 2017 American Academy of Paediatrics Paediatric Hypertension Guideline [1].

	Neonate and Infants	Children 1–13 Years Old	Children ≥ 13 Years Old
Normal		<90th percentile	<120/<80 mm Hg
Elevated BP	≥95th percentile for postmenstrual age	≥90th percentile to <95th percentile or 120/80 mm Hg * to <95th percentile	BP: 120/<80 to 129/<80 mm Hg
Stage 1 HTN:		≥95th percentile to <95th percentile + 12 mmHg, or 130–139/80–89 mm Hg *	130–139/80–89 mm Hg
Stage 2 HTN:		≥95th percentile + 12 mm Hg, or ≥140/90 mm Hg *	≥140/90 mm Hg

Blood Pressure (BP); Hypertension (HTN); * (whichever is lower).

**Table 3:** Secondary causes of hypertension in paediatric patients [4,10,11,12].

Systems	Neonates and Infants	Children	Adolescents
Pulmonary	Bronchopulmonary dysplasia	Obstructed sleep apnoea	Obstructed sleep apnoea
Cardiac	Congenital heart disease Coarctation of aorta	Coarctation of aorta	Coarctation of aorta
Neurological	Pain Intracranial hypertension Seizure Subdural haematoma	Increased intracranial pressure	Increased intracranial pressure
Reno-vascular	Thromboembolism Fibromuscular Dysplasia Renal artery stenosis Renal venous thrombosis Compression of renal artery Idiopathic arterial calcification	Fibromuscular Dysplasia Midaortic Syndrome Takayasu Arteritis Renal artery hypoplasia	Fibromuscular Dysplasia Midaortic Syndrome Takayasu Arteritis
Renal parenchymal disease	Congenital Polycystic kidney disease Ureteropelvic junction obstruction Acquired Acute tubular injury Cortical necrosis Obstruction (stones, tumours)	Glomerulonephritis Obstructive uropathy	Glomerulonephritis Obstructive uropathy
Endocrine	Congenital adrenal hyperplasia Hyperaldosteronism Hyperthyroidism Pseudo hypoaldosteronism type II	Cushing syndrome Hyperaldosteronism Hyperthyroidism	Cushing syndrome Hyperaldosteronism Hyperthyroidism
Neoplasia	Wilms tumour Mesoblastic nephroma Neuroblastoma Pheochromocytoma	Wilms tumour Neuroblastoma Pheochromocytoma	Pheochromocytoma
Medications/intoxications	Dexamethasone Adrenergic agents Vitamin D intoxication Theophylline Caffeine Pancuronium Phenylephrine Maternal Cocaine Heroin	Sympathomimetics: cocaine, amphetamines, pseudoephedrine Corticosteroids	Sympathomimetics: cocaine, amphetamines, pseudoephedrine Corticosteroids Oral contraceptive
Miscellaneous	Prematurity Closure of abdominal wall defect Uterine arterial and venous catheters Low birth weight Extracorporeal membrane oygenation		Primary hypertension Preeclampsia/eclampsia
Genetic syndromes	Von Hippel-Lindau (VHL gene) MEN 2 (RET & SDH genes) Neurofibromatosis type 1 (NF1 gene)		

**Table 4:** Antihypertensive medications commonly used in intraoperative paediatric hypertensive crises [11,14].

Drug Class	Drug	Route	Dose	Onset of Action	Adverse Effects
First Line Agents					
Direct Vasodilators	Sodium Nitroprusside	Intravenous infusion	0.5–10 μg/kg/min	2–10 min	Thiocyanate toxicity, inactivated by light.
	Hydralazine	Intravenous bolus	0.2–0.6 mg/kg. Maximum single dose 20 mg	5–20 min	Reflex tachycardia, headache, fluid retention.
Beta Blockers	Esmolol	Intravenous infusion	100–500 μg/kg/min, up to 1000 μg/kg/min	2–10 min	Contraindicated in asthma, may cause bradycardia.
Calcium Channel Blockers (CCB)	Nicardipine	Intravenous bolus/infusion	30 μg/kg up to 2 mg/dose/0.5–4 μg/kg/min	Within minutes	Reflex tachycardia.
Second Line Agents					
Direct Vasodilators	Diazoxide	Intravenous bolus	1–3 mg/kg every 5–15 min	Within minutes	Risk of hypotension in large doses.
	Nitroglycerine	Intravenous infusion	0.1–2 μg/kg/min	1–2 min	Methemoglobinemia, vasodilating effect primarily on the venous side, efficient in heart failure, limited efficacy in children.
Alpha and Beta Blockers	Labetalol	Intravenous bolus/infusion	0.2–1 mg/kg/dose, up to 40 mg/dose/0.25–3 mg/kg/h	2–5 min	Contraindicated in asthma, heart failure, bradycardia.
Alpha Blockers	Phentolamine	Intravenous bolus	0.05–0.1 mg/kg/dose, up to 5 mg	Immediate	Tachycardia. Used only in pheochromocytoma.
Angiotensin-Converting Enzyme Inhibitors (ACEIs)	Enalaprilat	Intravenous bolus	5–10 μg/kg/min, up to 1.2 mg/dose	15 min	Contraindicated in suspected bilateral renal artery stenosis.
Diuretics (Loop)	Furosemide	Intravenous bolus	0.5–5 mg/kg/dose	Within minutes	
